# Emotional Ego- and Altercentric Biases in High-Functioning Autism Spectrum Disorder: Behavioral and Neurophysiological Evidence

**DOI:** 10.3389/fpsyt.2022.813969

**Published:** 2022-02-18

**Authors:** Helena Hartmann, Lukas Lengersdorff, Hannah H. Hitz, Philipp Stepnicka, Giorgia Silani

**Affiliations:** ^1^Social, Cognitive and Affective Neuroscience Unit, Department of Cognition, Emotion, and Methods in Psychology, Faculty of Psychology, University of Vienna, Vienna, Austria; ^2^Department of Clinical and Health Psychology, Faculty of Psychology, University of Vienna, Vienna, Austria

**Keywords:** self-other distinction, Cyberball, emotional egocentricity bias, emotional altercentricity bias, autism (ASD), fMRI

## Abstract

Self-other distinction is a crucial aspect of social cognition, as it allows us to differentiate our own mental and emotional states from those of others. Research suggests that this ability might be impaired in individuals on the autism spectrum, but convincing evidence of self-other distinction difficulties in the emotional domain is lacking. Here we aimed at evaluating emotional self-other distinction abilities in autistic and non-autistic adults, in two behavioral pilot studies and one fMRI study. By using a newly developed virtual ball-tossing game that induced simultaneous positive and negative emotional states in each participant and another person, we were able to measure emotional egocentric and altercentric biases (namely the tendency to ascribe self-/other-related emotions to others/ourselves, respectively). Despite no behavioral differences, individuals on the autism spectrum showed decreased activation (1) in the right temporoparietal junction (rTPJ) during active overcoming of the emotional egocentric bias vs. passive game viewing, and (2) in the right supramarginal gyrus (rSMG) during ego- vs. altercentric biases, compared to neurotypical participants. These results suggest a different recruitment of these two regions in autistic individuals when dealing with conflicting emotional states of oneself and another person. Furthermore, they highlight the importance of considering different control conditions when interpreting the involvement of rTPJ and rSMG during self-other distinction processes.

## Introduction

Social cognition, the capacity to sense, represent and judge our own social behaviors and those of others, is an ubiquitous aspect of the human mind and crucial for everyday social interactions [see (1) for a review]. Humans represent and infer other's mental states—an ability known as mentalizing or Theory of Mind—using multiple self- related processes, e.g., when putting oneself in the shoes of another person [(2–4) for reviews]. At the same time, adequate social behavior demands that we can distinguish between self- and other related representations, a crucial cognitive skill termed self-other distinction [(5, 6) for reviews; (7, 8)].

The importance of self-other distinction becomes evident in cases where it breaks down. For example, it has been proposed that this ability is disrupted or at least qualitatively different in individuals on the autism spectrum, a neurodevelopmental condition largely characterized by impairments regarding social communication and social interaction (9). Autistic individuals, for example, are less likely to draw a sharp distinction between their own and another's perspective [(10) for a review], and are more likely to confuse self- and other-related emotions [(11) for a review; (12) for a meta-analysis; (13)], leading to distorted cognitive and affective representations named biases. Deschrijver and Palmer (14) have recently suggested that insufficient monitoring of mental conflicts—i.e., when mental states differ between oneself and another person, might be the main reason behind the aforementioned difficulties in autistic individuals, rather than the general lack of mental representation abilities or “mindblindness”. Similarly, Bird and Viding (10) speculate that the so-called “self-other switch” could be impaired in autistic individuals, who might, in turn, be more or less affected by others' emotional states. This is in line with studies relating the ability to accurately represent self and others' mental and emotional states to personal distress in individuals on the spectrum (13).

In spite of the abundance of studies on increased cognitive biases in autism, using e.g., false belief or social discounting tasks [(15, 16) for a review; (17, 18)], biases in the emotional domain seem to have gotten less attention over the years, in spite being a major problem for this population [(14) for a recent review; (19, 20)]. Our study aimed to fill this gap by investigating the existence of such affective biases in autistic and non-autistic individuals and relating them to possible neurophysiological mechanisms.

To date, only one other study (21) was performed to assess emotional self-other distinction in autism spectrum conditions. Using a visuo-tactile task where participants rated the (un)pleasantness of different objects simultaneously touching themselves and another participant, the authors observed preserved behavioral self-other distinction (but not mentalizing abilities) in autistic compared to matched NT individuals. Furthermore, in an independent pool of autistic individuals, they observed reduced resting state connectivity in the brain mentalizing network (i.e., right temporoparietal junction; rTPJ), but intact network connectivity of the right supramarginal gyrus (rSMG), a region which plays a crucial role in overcoming those biases (6, 22, 23). Despite being the first of its type, this previous study suffered from three shortcomings that we aimed to address here: Firstly, the study did not clearly separate *egocentric* (i.e., the tendency to project one's own states onto others) from *altercentric* (i.e., the tendency to absorb another's state onto one's own) biases, which may have led to them canceling each other out, resulting in visibly preserved self-other distinction. Secondly, participants were tested in their pleasantness evaluation of simple touch stimuli, but the question remains whether the same holds true for more complex emotions evident in daily social interactions. Lastly, the study could not establish a direct link between behavior and brain as independent samples were used for either analysis, making it difficult to draw conclusions involving both.

In order to replicate and extend this important initial work, we employed a newly developed version of the *Cyberball* task [a virtual ball-tossing game; (24, 25)], combined with functional Magnetic Resonance Imaging (fMRI) assessment. The task was designed to induce simultaneous positive and negative emotional states in a participant and another person by means of inclusion and exclusion from the game. Participants either took the role of active player or passive observer in the game, judged either their own emotional state or the one of the (spatially) opposite player and could either be in a congruent or incongruent emotional state (elicited through game inclusion or exclusion) with that other person. In short, the task entailed three different conditions: *self active* (= judging yourself as an active player), *other active* (= judging another person as an active player), and *other passive (*= judging another person but from a neutral, outside perspective as a passive observer). While the first two conditions were employed to resemble previous operationalizations of emotional egocentric biases [i.e., altercentric biases are subtracted from egocentric biases; (23)]; the last condition was introduced to overcome the limitation of such operationalization (namely the impossibility to assess the egocentric and altercentric biases independently). By using an emotionally neutral condition, where participants are asked to judge other emotions without being themselves directly involved, we could control for task complexity (i.e., incongruency between different emotional states) without canceling out the processing of self-other representations, and therefore capturing the emotional egocentric bias in a cleaner way.

The new task was first validated in two behavioral studies with independent NT samples (*n*'s = 45 and 52, see Supplementary Material) and then applied in our fMRI study, testing 21 NT and 21 autistic individuals. On the behavioral level, we hypothesized higher emotional egocentric biases (EEB) and/or emotional altercentric biases (EAB) in autistic individuals compared to NTs, but no group differences during passive observation. In other words, we predicted lower self-other distinction abilities in individuals on the autism spectrum compared to NTs, indicated by stronger ego- and altercentric judgment shifts when dealing with concurrent incongruent compared to congruent emotional states. As previous studies had used the EAB as a control, here we were additionally interested in differences between EEB and EAB. On the neurophysiological level, we hypothesized reduced activity in areas related to self-other distinction, such as rTPJ and rSMG in autistic compared to NT individuals during two active playing conditions measuring EEB and EAB, but no group differences in the passive condition.

## Methods

### Statistical Analysis

The manuscript was checked for reporting errors in the statistical analyses using statcheck [http://statcheck.io/, (42)] and for reference errors using reciteworks (4cite Labs, https://reciteworks.com/).

### Open Data and Materials Statement

Unthresholded statistical maps of the fMRI data are uploaded on NeuroVault (https://neurovault.org/collections/11646/). The here used version of the Cyberball task can be shared upon request.

### Participants

In all three studies, NT participants were recruited by means of poster and online advertisements as well as an online participant recruitment system of the University of Vienna. Participants indicated *via* a thorough online questionnaire that they had no past or present psychiatric or neurological illnesses (NT sample only), had not taken part in similar studies before, had never studied psychology and had no risk factors concerning MR scanning (the latter criterion for the fMRI study only). In the fMRI study, autistic participants were recruited *via* an existing database of participants that registered in the past for taking part in university studies, as well as by contacting institutions that diagnose and treat people on the autism spectrum in Vienna and Linz. For all autistic participants, we confirmed a clinical ICD-10/DSM-IV diagnosis of the Asperger syndrome (F84.5/299.80, nowadays subsumed under “autism spectrum disorder”) given by an accredited institution, and if possible assessed with the Autism Diagnostic Observation Schedule (26). Twenty-one autistic participants were matched to 21 NT participants regarding gender, age, handedness, and intelligence (see Table 1 for sample characteristics and matching criteria). Six autistic participants indicated having comorbidities such as depression or anxiety. A *post hoc* sensitivity power analysis in G^*^Power [version 3.1.9.2; (27)] showed that with our study we could have reasonably been able to detect a minimum effect size of Cohen's *d* = 0.40 (α = 0.05, 1-β = 0.80).

**Table 1:** Sample characteristics and matching criteria in the fMRI study.

	**Neurotypical**	**Autistic**
**Nationality**		
Austria Germany	16 5	19 2
Gender^*^		
male	16	15
female	5	6
Medication intake^**^		
Yes	1	7
No	20	14
Handedness^*^		
Right	20	20
Left	1	1
Age^*^	36.14 (10.16)	36.52 (10.07)
Intelligence^*^		
SPM	7.24 (1.79)	7.62 (1.28)
MWT-B	30.00 (4.34)	29.57 (4.00)

*Sociodemographic data of the participants in the fMRI study. Frequencies regarding nationality, gender, and handedness as well as averages (standard deviations) for age and intelligence are given*.

* *Matching criteria;*

** *The neurotypical participant took thyroid and blood pressure medication, six autistic participants took medication against psychiatric disorders such as e.g., depression, anxiety, or panic disorder, and one autistic participant took hormonal birth control; SPM, Standard Progressive Matrices; MWT-B, Mehrfachwahl-Wortschatz-Intelligenztest (version B)*.

### Questionnaires

For matching purposes, two short forms of measures targeting general intelligence were given to all participants in the fMRI study. The Standard Progressive Matrices [SPM; (28)] is a non-verbal measure to assess general intelligence with figural material. The version used here contained nine items in each of which participants had to select the missing piece to an incomplete pattern. The Mehrfachwahl-Wortschatz-Intelligenztest [MWT-B; (29)] measures crystallized intelligence. For each item, participants needed to choose which one out of five given words is an existing word. We further assessed the following personality questionnaires: The degree to which a person reports traits associated with the autistic spectrum were measured using the 33-item version of the Autism-Spectrum-Quotient [AQ-k; (30)]. Those traits are based on the triad of impairments (social communication, social interaction and restricted, repetitive behaviors or activities) and on other areas of cognitive abnormality. They are viewed as part of a quantitative continuum where a person can be located based on their score. The cut-off score for a clinically significant level of autistic traits is ≥ 17. Alexithymia, the subclinical inability to identify and describe emotions, was measured with the 20-item version of the Toronto-Alexithymia-Scale [TAS-20; (31, 32)] and is divided into three subscales (Difficulty Identifying Feelings = DIF, Difficulty Describing Feelings = DFF, Externally-Oriented Thinking = EOT). To measure the subjective empathic qualities in the participants, the Interpersonal-Reactivity-Index (IRI) was used (33, 34). This multidimensional self-report questionnaire incorporates affective as well as cognitive aspects of the empathic reaction and is divided into four subscales, three measuring aspects of affective (Fantasy, Empathic Concern and Personal Distress) and one measuring cognitive empathy (Perspective Taking). Finally, the revised version of the Beck-Depression-Inventory [BDI-II; (35)] was used to measure the amount of depressive symptoms. All questionnaires were administered *via* Paper and Pencil format in their German versions. Cohen's *d*'s for questionnaire comparisons were calculated using the effect size calculation spreadsheet (version 4.2) provided by Lakens (36).

### Procedure

In all three studies, each participant gave written consent at the outset of their respective session. The overall project was approved by the ethics committee of the Medical University of Vienna (EK 1166/2015) and performed in line with the latest revision of the Declaration of Helsinki (2013) on ethical principles for medical research involving human participants. The procedure of the two behavioral pilot studies can be found in the Supplementary Material. For the fMRI study, each participant was asked not to take any medication, drugs, or alcohol 24 hours prior to the appointment. The task procedure was changed slightly to create a social situation adapted to the scanner environment. Participants came together with four confederates (always two males and two females) who were presented as other participants, but in fact were helpers of the experimenters merely acting as participants. This was done to make the social situation of the virtual ballgame more realistic and to make the participants believe that they were playing the game together with other participants. The five participants were given verbal instructions and played two short practice rounds on computers in the control room of the MRI scanner. The confederates were asked to remain seated and told they would be playing the main game from outside the scanner, while the participant was led into the scanner room. After general adjustments, each participant played three runs of the Cyberball game. After the task, the structural scan was acquired. When participants left the scanner room, they were asked to fill out additional questionnaires and told that the other participants had already left. Before leaving, each participant received 25 Euros as compensation and was fully debriefed by the experimenters about any deception used in the study, specifically to avoid negative emotions regarding exclusion by other individuals in the game. The whole procedure lasted around 2.5 h.

### Cyberball Task

**Figure 1:**
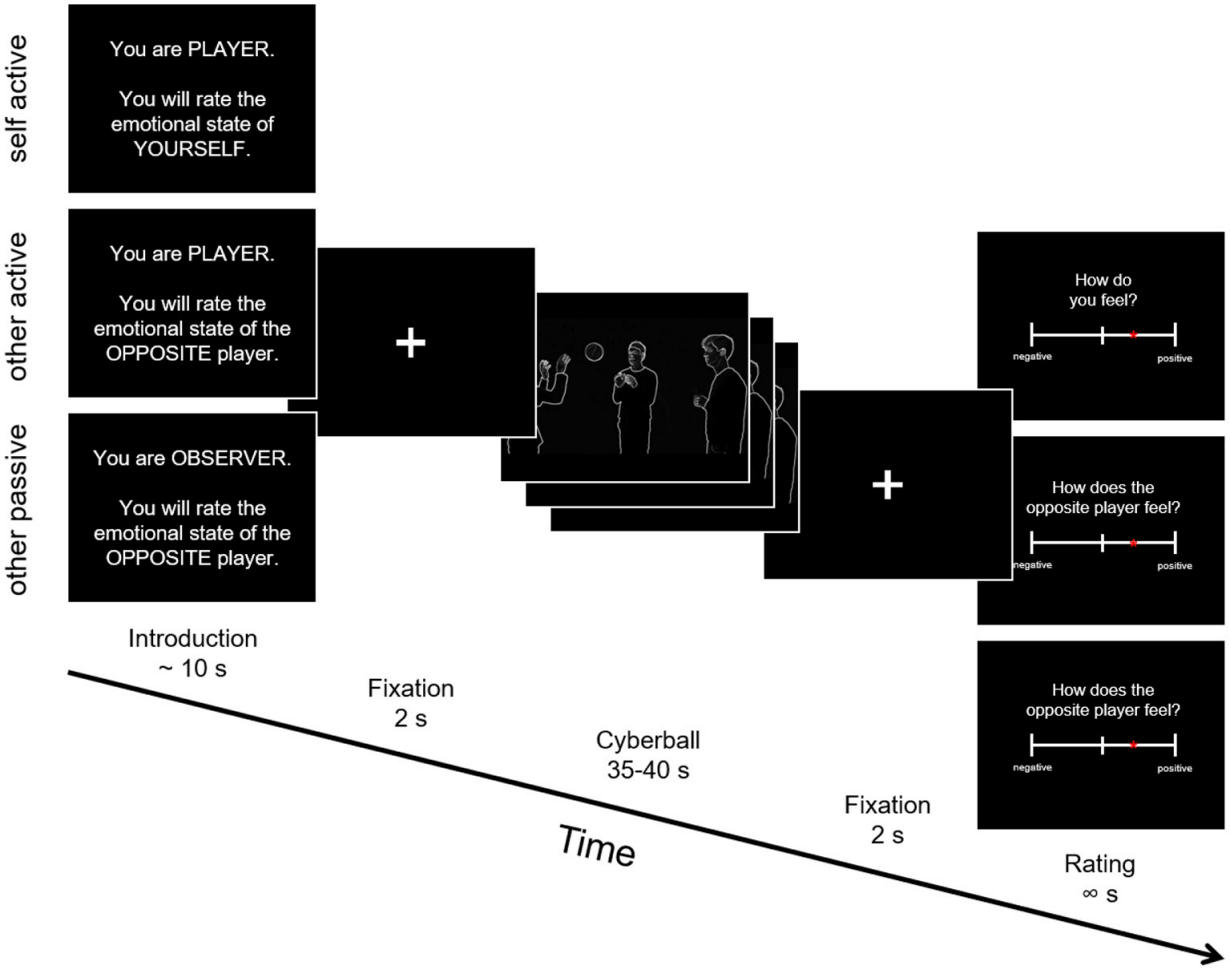
Schematic depiction of the Cyberball trial structure. Participants were shown an introduction screen with information whether they would be an active player or observer in the next game and whether they would judge the emotional state of themselves or of the player standing opposite of them. Following that was the Cyberball gameplay between 35 and 40 seconds, where different movie clips were shown depending on the left and right ball-throws of the participants. After each trial, emotional state ratings were collected.

Participants played a modified version of Cyberball, a virtual computer-based ball-tossing game in all three studies (25, 37–40). We created an adapted version fundamentally similar to the version used in Novembre et al. (24), but using videos depicting white silhouettes of real humans on a black background^1^. Importantly, this version was designed to be used in the autistic population, ensuring as little distraction as possible by avoiding for example facial expressions and uneven background (see Figure 1). During the game, four avatars supposedly controlled by participants interacted in a virtual environment by throwing a ball to each other. The three main conditions differed in respect to whether: (a) the participant was actively involved in the game (*self active, other active*) or only observing the others play (*other passive*), and (b) whether the participants were asked to rate their own feelings (*self active*) or those of the player standing opposite of them (*other active, other passive*).

Every condition included 16 trials of ball-tossing with ~13–15 ball throws per trial. During inclusion trials, the participant (the self) receives the ball and can throw it back to one of the others. Throwing was realized by pressing the arrow keys in the behavioral studies or the buttons on an fMRI-compatible button-box in the fMRI study. During exclusion trials, the player starts in the first trial with throwing the ball but then does not receive it back for the rest of that trial. Furthermore, the congruency to the emotional state of the (spatially) opposite person was varied *via* inclusion or exclusion of self and other in the game^2^. During incongruent trials, one was excluded from the game while the other was excluded (i.e., self and other experiencing different emotions, either Self Included/Other Excluded or Self Excluded/Other Included). To measure self- and other-related emotional responses after each trial, participants were asked to rate either their own feelings (*self active*) or those of the person standing opposite of them (*other active* and *other passive*) on a continuous rating scale from negative to positive (representing exact values between −250 and +250, later rescaled to values between −10 and +10), staying on the screen until the response was acquired. The game was played in a fixed, previously pseudorandomized trial order in all studies and all participants. In the fMRI study, the three conditions were played in the scanner in separate runs, in one of six pseudorandomized orders. All videos shown in the game were filmed with a Panasonic Lumix DMC-FZ200 and later edited with Windows Movie Maker (Microsoft; applied setting: edge detection) and Corel VideoStudio Pro X8 (https://www.videostudiopro.com/en/pages/videostudio-x8/; applied filters: invert and monochrome) to a final resolution of 640 ×360 pixel. The Cyberball task was implemented in MATLAB 2010b (41), using Cogent 2000 (Version 1.29, http://www.vislab.ucl.ac.uk/cogent_2000.php).

### fMRI Data Acquisition

Functional data of the fMRI study was acquired using a 3 Tesla Siemens Magnetom Skyra MRI-system (Siemens Medical, Erlangen, Germany) in Vienna, equipped with a 32-channel head coil as well as a high-performance gradient system for fast, high-resolution whole brain multiband echoplanar imaging. The scanning sequence included the following parameters: Echo time (TE)/repetition time (TR) = 34/704 ms, flip angle = 50°, interleaved multi-slice mode, interleaved acquisition, field of view = 210 mm, matrix size = 96 × 96, voxel size = 2.2 × 2.2 × 3.5 mm^3^, 32 axial slices coplanar the connecting line between anterior and posterior commissure, and slice thickness = 3.5 mm. For each of the three conditions, functional volumes were acquired within 11–13 mins durations per run (the exact number of volumes depending on the choice of ball throws and the rating velocity of the participants), with small breaks in between the runs. Structural images were acquired using a magnetization-prepared rapid gradient-echo sequence (TE/TR = 2.29/2,300 ms, ascending acquisition, single shot multi-slice mode, 176 sagittal slices, voxel size = 0.9 × 0.9 × 0.9 mm^3^, flip angle = 8°, slice thickness = 0.94 mm, field of view = 240 mm).

### Behavioral Data

For the behavioral analyses, our aim was to investigate EAB and EEB as well as possible group differences in the fMRI study. Subjective ratings pertaining to the different conditions were entered in an analysis of variance (ANOVA) using the R package ezANOVA including the within-subjects factors *target* (self active, other active, other passive), *congruence* (congruent, incongruent), and *valence* (positive, negative), and the between-subjects factor *group* (autistic, NT). As in Silani et al. (23), ratings in the unpleasant conditions were multiplied by −1 to allow the comparison of the bias across valences. All behavioral analyses were done in RStudio [version 4.1.0; (43)]. While we focus on the results of the fMRI study in the present manuscript, the analysis of the two behavioral pilot studies can be found in the Supplementary Material.

### Brain Data

#### Preprocessing and Analysis

To preprocess and statistically analyze the fMRI data, the current version of the software Statistical Parametric Mapping (SPM12, Wellcome Trust Centre for Neuroimaging, https://www.fil.ion.ucl.ac.uk/spm/software/spm12/) running on MATLAB Version R2010b (41) was used. Preprocessing included realignment, coregistration of structural and functional images, segmentation into gray matter, white matter and cerebrospinal fluid, spatial normalization, and spatial smoothing by convolution with an 8 mm full-width at half-maximum (FWHM) Gaussian kernel. The first-level design matrix of each subject contained two separate regressors (playing time during trial and rating) for each of the four conditions (Self Included/Other Included, Self Included/Other Excluded, Self Excluded/Other Excluded, Self Excluded/Other Included), leading to a total of eight regressors for each of the three targets (self active, other active, other passive) and 24 regressors in total. In general, the full playing time of the ball game in each trial was modeled in a block-design fashion and convolved with SPM12's standard canonical hemodynamic response function. Nuisance regressors included six realignment parameters and an additional two modeling (mean-centered) white matter and cerebrospinal fluid signal for each run [the latter two were extracted using the REX toolbox by (44)]. For the group level, we created three contrast images incorporating the three conditions by contrasting congruent and incongruent trials and averaging over valence: EAB_active_ = self active (incongruent > congruent), EEB_active_ = other active (incongruent > congruent), and EEB_passive_ = other passive (incongruent > congruent). This averaging over valence for all analyses of the fMRI data was done purposefully as the stimuli shown for each valence (inclusion and exclusion) differed in important aspects such as motor movement and activity which would have influenced brain activity. For example, participants were only able to conduct one ball throw during exclusion trials while they were constantly throwing the ball in inclusion trials. This made an interpretation of the contrasts involving valence difficult.

#### Whole Brain Analysis

For the whole-brain analysis, the second-level model included a flexible-factorial design with the within-subject factors *subject* and *condition*, and the between-subjects factor *group* (autistic vs. NT). As a proof of concept and to evaluate whether our manipulation of congruency was successful, we first report the mean contrasts for each of the three conditions over both groups. To test our main hypotheses of increased EAB and EEB in the autistic participants, we then report the group comparisons for each of the three conditions (EAB_active/NT_ vs. EAB_active/autistic_, EEB_active/NT_ vs. EEB_active/autistic_, EEB_passive/NT_ vs. EEB_passive/autistic_, as well as the complementary contrasts for autistic vs. neurotypical). Finally, we report group comparisons for EEB_active_ after subtracting either EAB_active_ [as done in the previous study by (23)] or EEB_passive_ (our new control condition) as well as the additional contrast in our region-of-interest (ROI) analyses (see below): EEB_active_ + EEB_passive_ vs. EAB_active_, both for NT vs. autistic individuals and vice versa. All whole brain imaging results are reported at a cluster probability of *p* < 0.05 [familywise-error (FWE)-corrected, cluster-forming threshold of *k* = 279, initial cluster-defining threshold *p* < 0.001 uncorrected]. Anatomical regions were labeled with SPM12's Anatomy toolbox [version 2.2c; (45)].

#### Region of Interest Analyses

Lastly, to explore differences in two major regions identified as being involved in social cognition, self-other distinction, and emotional egocentricity, we conducted ROI analyses in rSMG and rTPJ. We created 8 mm spheres around two peak MNI coordinates taken from the independent study that also investigated self-other distinction in autistic and non-autistic participants using the visuo-tactile egocentricity paradigm (21): rSMG: x = 65, y = −37, z = 33; rTPJ: x = 51, y = −52, z = 21, using MarsBaR for ROI creation (46). We then extracted parameter estimates for each of the two ROIs using the first-level contrast images of each participant, separate for congruence and target (averaging over valence) with REX (44). We then calculated an ANOVA including the within-subjects factors *target* (self active, other active, other passive), *congruence* (congruent, incongruent), *roi* (rSMG, rTPJ) and the between-subjects factor *group* (autistic, NT). We focused these analyses on two aspects inherent in our task: (1) Checking for group differences in emotional egocentricity related to the degree of emotional involvement by contrasting the other active and other passive condition, and (2) checking for differences in ego- vs. altercentric judgments by contrasting all other-related (other active and other passive) with the self-related condition (self active). Mauchly's test for sphericity was not statistically significant for any effects including the factor target, thus fulfilling the sphericity assumption.

## Results

### Questionnaire Results

In the personality trait questionnaires, the two groups differed significantly in their reported autistic, alexithymic and depressive traits, with autistic subjects consistently indicating higher values than NT (see Table 2 for an overview). Regarding empathic abilities, autistic participants described significantly greater personal distress, while NT ascribed themselves significantly higher perspective taking abilities. The two groups did not significantly differ regarding their trait empathic concern and fantasy.

**Table 2:** Differences between autistic and neurotypical participants in trait personality questionnaires in the fMRI study.

	**Neurotypical**	**Autistic**	***t***	***df***	***p***	**Cohen's *d***
**Autistic traits**	6.76 (4.48)	23.95 (5.62)	−10.96	40	<0.001	3.47
**Alexithymia**						
DIF	12.29 (4.45)	20.14 (5.70)	−4.98	40 40	<0.001	1.57
DDF	11.47 (3.68)	17.43 (4.04)	−4.99	40	<0.001	1.58
EOT	16.09 (4.11)	20.52 (4.86)	−3.19		0.003	1.01
**Empathy**						
Fantasy	3.11 (0.68)	2.68 (0.85)	1.80	40	0.079	0.57
Empathic Concern	3.66 (0.66)	3.37 (0.76)	1.33	40	0.189	0.42
Personal Distress	2.51 (0.43)	3.09 (0.79)	−2.99	40	0.004	0.93
Perspective Taking	3.69 (0.49)	3.04 (0.66)	3.61	40	<0.001	1.15
**Depression**	6.24 (6.30)	13.29 (10.01)	−2.73	40	0.009	0.86

*Trait questionnaire data of the fMRI study separated by group. Average item sum scores (autism, alexithymia, depression) or average item scores (empathy) plus standard deviations in brackets. DIF, Difficulty Identifying Feelings subscale; DDF, Difficulty Describing Feelings subscale; EOT, Externally Oriented Thinking subscale*.

### Behavioral Task Results

**Figure 2:**
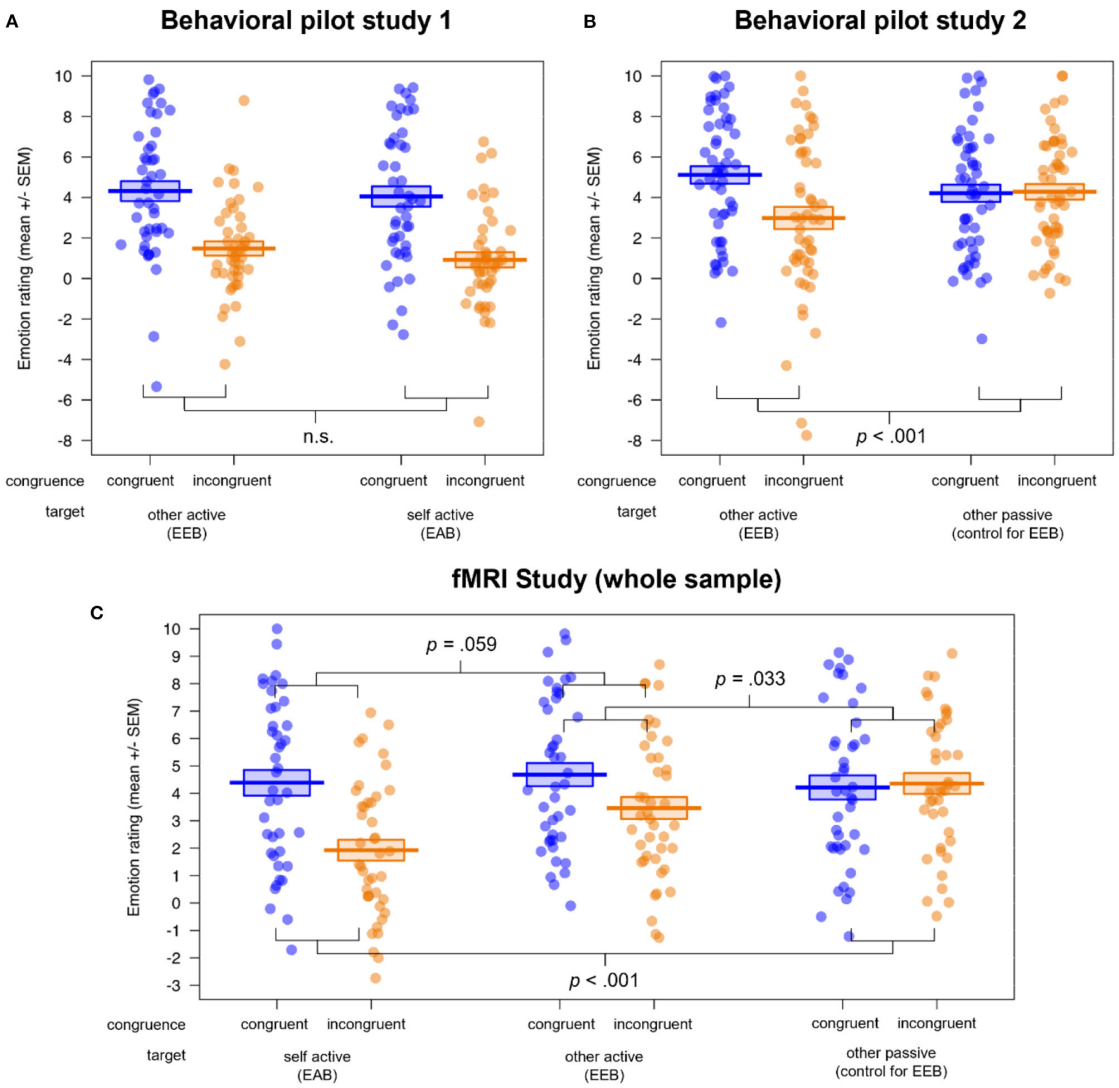
Visualization of the behavioral results in **(A)** behavioral pilot study 1 (*n* = 45), **(B)** behavioral pilot study 2 (*n* = 52) and **(C)** the fMRI study (*n* = 42). All studies showed a successful induction of emotional biases (EEB and/or EAB), visible in the rating differences for congruent vs. incongruent emotional states. While the congruence × target interactions were significant in behavioral pilot study 2 and the fMRI study, behavioral pilot study 1 showed no difference between the magnitudes of the EEB and EAB. Additionally, behavioral pilot study 2 and the fMRI study validated the passive viewing condition as a control condition for the EEB. EEB, emotional egocentricity bias; EAB, emotional altercentricity bias.

The results of the behavioral pilot study data can be found in the Supplementary Material (see also Supplementary Tables S1, S2). These analyses revealed conclusive evidence that our version of the Cyberball task was able to manipulate the congruency of simultaneous emotional states, as we observed an EEB and EAB in the participants' ratings in behavioral pilot study 1, indexed by significant main effects of congruence and target × congruence interactions (all *p*'s ≤ 0.001). Furthermore, we established the third condition, other passive, as a valid control condition for the EEB, as no difference between congruent and incongruent condition (i.e., no bias) was observed there in behavioral pilot study 2 (see also Figures 2A,B)^3^.

In the ANOVAs of the fMRI study rating data (see Figure 2C here and Supplementary Table S3), we observed main effects of congruence (*F*_(1, 40)_ = 18.06, *p* < 0.001, η^2^ = 0.031) and valence (*F*_(1, 40)_ = 4.57, *p* = 0.039, η^2^ = 0.021), with more extreme ratings for congruent (*M* ± *SD* = 4.43 ± 3.49) compared to incongruent (*M* ± *SD* = 3.25 ± 3.47) emotional states, and for negative (*M* ± *SD* = 4.31 ± 3.55) compared to positive (*M* ± *SD* = 3.36 ± 3.44) emotional states of the person to be judged. Furthermore, a main effect of target (and follow-up *post hoc* comparisons with the function pairs) indexed that the ratings in the self active (*M* ± *SD* = 3.16 ± 3.68) differed significantly from those in the other active (*M* ± *SD* = 4.07 ± 3.53; *p* = 0.044) as well as the other passive condition (*M* ± *SD* = 4.29 ± 3.26; *p* = 0.009), independent of congruence or valence (*F*_(2, 80)_ = 13.36, *p* < 0.001, η^2^ = 0.022).

A significant target × congruence (*F*_(2, 80)_ = 13.43, *p* < 0.001, η^2^ = 0.026; and follow-up *post hoc* comparisons with the function pairs) showed that the difference in congruent vs. incongruent ratings, i.e., the emotional bias, was significantly lower for the other passive (control for EEB; *M*_*diff*_ = 0.14) compared to the self active (EAB; *M*_*diff*_ = −2.46; *p* < 0.001) as well as compared to the other active condition (EEB; *M*_*diff*_ = −1.22; *p* = 0.033). Mirroring the results of behavioral pilot study 1, there was no significant difference between EAB and EEB (*p* = 0.059). We also observed a group × target interaction (*F*_(1, 40)_ = 3.24, *p* = 0.044, η^2^ = 0.005), showing that individuals on the autism spectrum had more extreme ratings compared to NTs in the self active (*M*_*diff*_ = 1.68; *p* = 0.021), but not in the other active (*M*_*diff*_ = 0.64; *p* = 0.535) or the other passive condition (*M*_*diff*_ = −0.06; *p* = 0.925). Lastly, we found a target × congruence × valence interaction (*F*_(2, 80)_ = 4.85, *p* = 0.010, η^2^ = 0.004), which showed that the rating difference between congruent and incongruent as well as positive and negative emotional states was highest for self active (*M* ± *SD* = 2.08 ± 4.08), medium for other passive (*M* ± *SD* = 0.99 ± 4.52) and lowest for other active (*M* ± *SD* = −0.03 ± 5.18). Importantly, an absence of any other group effects and specifically no significant congruence × group or congruence × target × group interactions (all *p*'s > 0.075) showed that both groups were equally susceptible to emotional biases on the behavioral level.

### Whole Brain Results

**Figure 3:**
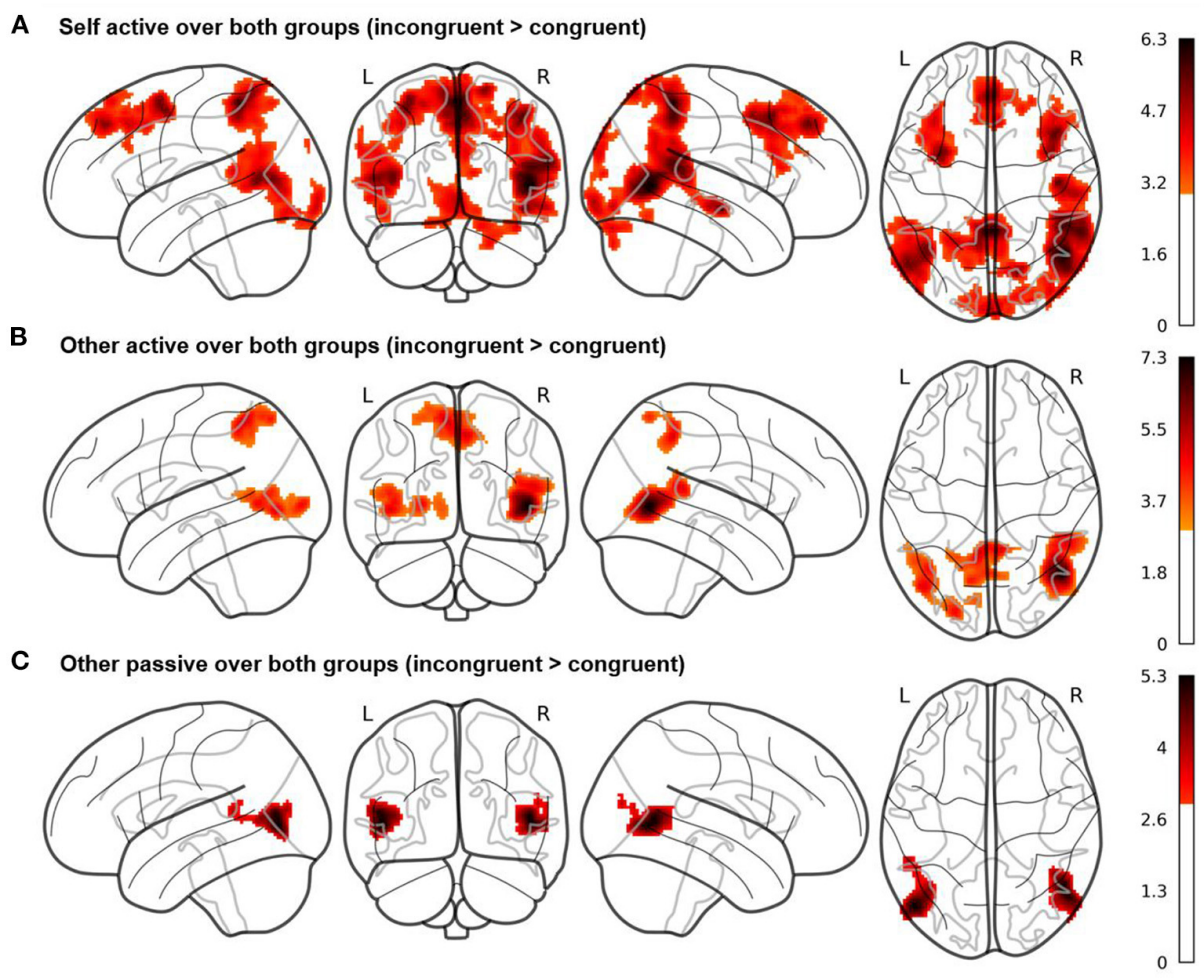
Whole brain results of the fMRI study for the three Cyberball conditions **(A)** self active, **(B)** other active, and **(C)** other passive. Contrasts are averaged over the factors valence and group, calculated as incongruent > congruent conditions and displayed at a cluster probability of *p* < 0.05 (familywise-error (FWE)-corrected, cluster-forming threshold of *k* = 279, initial cluster-defining threshold *p* < 0.001 uncorrected). The results show activity in brain regions such as precuneus and superior temporal gyrus for the self active and other active conditions, while these regions are not active in the other passive condition.

In our whole brain analyses, we evaluated the three target contrasts over both groups (see Figure 3 here and Supplementary Tables S4–S6). In the mean contrast for self active (EAB) over both groups, we observed, among others, increased hemodynamic activity in bilateral middle temporal, frontal and superior medial gyri, right superior temporal gyrus, right superior parietal lobule, bilateral angular gyrus, right precuneus and right inferior frontal gyrus (see Figure 3A). Activity in these regions was increased during incongruent compared to congruent trials, when participants played the game and were asked to judge their own emotional state.

In the mean contrast for other active (EEB) over both groups (incongruent compared to congruent trials), we observed increased hemodynamic activity within the right middle and superior temporal gyri, left middle occipital gyrus and bilateral precuneus including the left superior parietal lobule (see Figure 3B). Activity in these regions was increased during incongruent compared to congruent trials, when participants played the game but judged the other's emotional state.

In the mean contrast for other passive over both groups, we observed increased hemodynamic activity in bilateral middle and left superior temporal gyri as well as middle occipital gyrus (see Figure 3C). Activity in these regions was increased during incongruent compared to congruent trials, when participants were again asked to judge the other's emotional state but were passively watching the game as an observer, while four others were playing.

However, evaluating our main hypothesis for group differences in the self active, other active and other passive conditions (either the individual conditions or as a differential contrast for EEB_active_–EAB_active_ or EEB_active_–EEB_passive)_, we did not observe any increased hemodynamic activity in either of the two groups. In fact, none of our whole brain group comparisons showed any active clusters at an FWE-corrected threshold. As we were specifically interested in the differential roles of two specific brain regions previously shown to be involved in self-other distinction, rTPJ and rSMG, we went on to analyse extracted brain activity in these areas.

### Region of Interest Results

**Figure 4:**
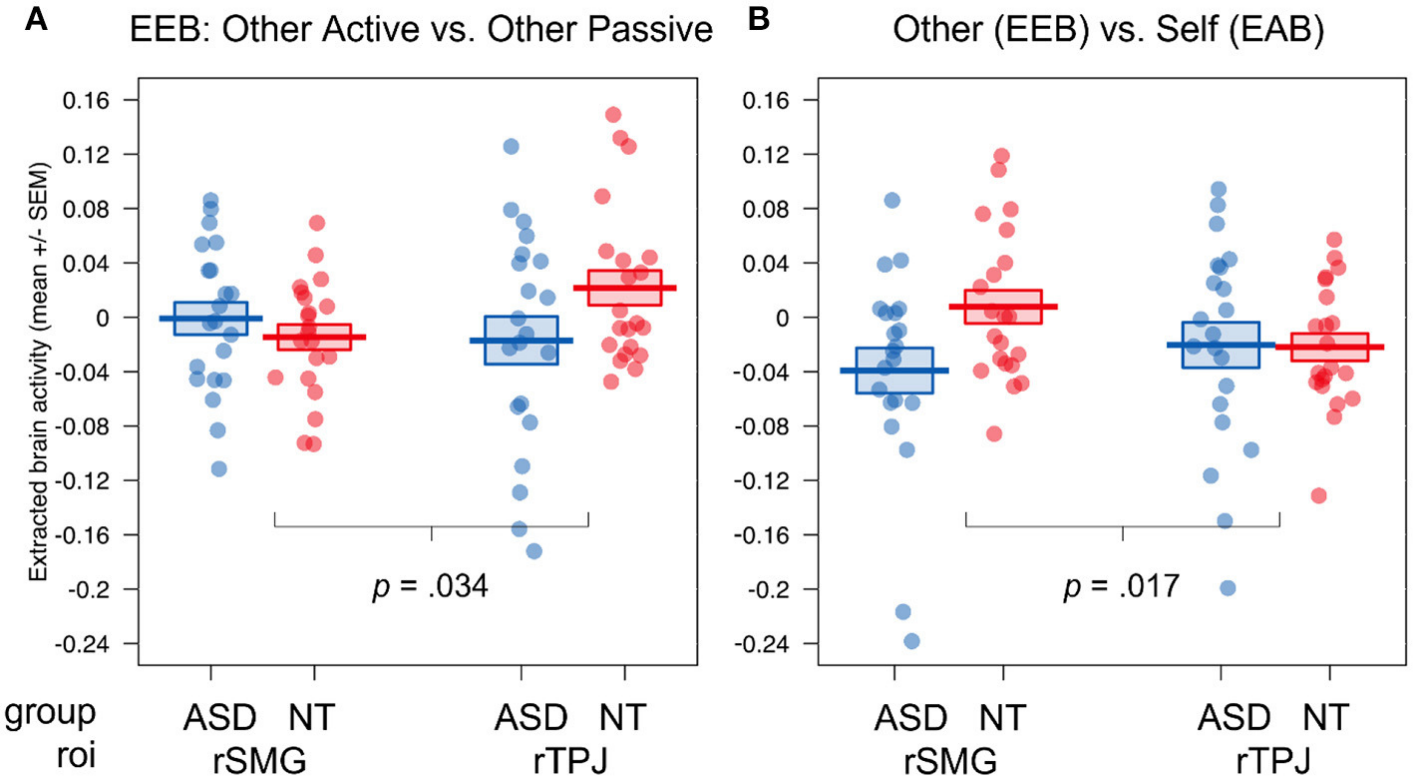
ROI results of the fMRI study for the group comparison of autistic vs. NT. Visualization of the four-way interaction group × congruence × target × roi, **(A)** the comparison of emotional involvement during egocentric judgements (other active vs. other passive), **(B)** the comparison of ego- (other active + other passive) vs. altercentric judgement conditions (self active); Displayed brain activity is averaged over the factors valence and calculated as incongruent—congruent; EEB, emotional egocentricity bias; EAB, emotional altercentricity bias; SEM, standard error of the mean; rTPJ, right temporoparietal junction; rSMG, right supramarginal gyrus.

In our ROI analysis, evaluating group differences in extracted brain activity in rSMG and rTPJ, we observed a main effect of congruence, showing increased activity in incongruent (*M* ± *SD* = −0.02 ± 0.008) compared to congruent (*M* ± *SD* = −0.03 ± 0.007) situations, independent of the target, roi or group membership: *F*(1, 42) = 16.89, *p* < 0.001, η^2^ = 0.005 (see Figure 4 here and Supplementary Table S7 for the full ANOVA results). We also found a main effect of roi, showing increased activity for rTPJ (*M* ± *SD* = 0.02 ± 0.008) compared to rSMG (*M* ± *SD* = −0.06 ± 0.007), independent of target, congruence, or group membership: *F*_(1, 40)_ = 29.07, *p* < 0.001, η^2^ = 0.11. Next, we observed a congruence × roi interaction, showing that the difference in activity between incongruent compared to congruent situations was increased in rTPJ (incongruent: *M* ± *SD* = 0.03 ± 0.01, congruent: *M* ± *SD* = 0.004 ± 0.01) compared to rSMG (incongruent: *M* ± *SD* = −0.07 ± 0.009, congruent: *M* ± *SD* = 0.06 ± 0.009), independent of target or group membership: *F*(1, 42) = 23.75, *p* < 0.001, η^2^ = 0.002.

Interestingly, we found a significant four-way interaction (group × congruence × target × roi, *F*_(2, 80)_ = 5.45, *p* = 0.006, η^2^ = 0.001). To follow up on this interaction, we calculated two orthogonal Helmert contrasts, contrasting (1) the degree of emotional involvement during emotional egocentricity (other active—other passive), and (2) ego- vs. altercentric conditions (0.5 ^*^ other active + 0.5 ^*^ other passive—self active) and compared the difference scores for incongruent—congruent situations for each roi between the two groups to follow up on the four-way interaction. This revealed that the difference in activity between other active and other passive conditions between the neurotypical and autistic group was increased in rTPJ compared to rSMG, with the NT group showing higher activity (*p* = 0.034; (see Figure 4A). In contrast, the difference in activity between the other- and self-related conditions between the neurotypical and autistic group was increased in rSMG compared to rTPJ, with the NT group showing higher activity (*p* = 0.017; see Figure 4B). In other words, while group differences in rTPJ activity seem to underpin evaluating egocentric judgements during active emotional involvement, group differences in rSMG relate to other- compared to self-related judgements. In both cases, participants on the autism spectrum had decreased activity in these regions compared to NTs.

## Discussion

The present study investigated self-other distinction and the occurrence of ego- and altercentric biases in autistic and non-autistic individuals.

Replicating and extending previous work on the topic (21), we were particularly interested in evaluating egocentric and altercentric biases independently, rather than one against the other, as both biases reflect difficulties with self-other distinction. To do this, we introduced an emotionally neutral condition, in which participants were asked to judge another person's emotions without being involved in the situation themselves. This condition served to control for task complexity (i.e., incongruency between different emotional states) without canceling out the processing of self- vs. other-related representations. Furthermore, we aimed to extend the work of Hoffmann et al. (47) to more complex emotions evident in daily social interactions by eliciting positive and negative feelings related to social inclusion and exclusion (48, 49). Lastly, we were interested to directly link behavioral group differences to differences in neural activation, by means of fMRI investigation.

On the behavioral level, the two behavioral pilot studies as well as the fMRI study confirmed the validity of our newly adapted Cyberball version. In all three studies, when individuals were actively playing the game and had to deal with incongruent compared to congruent emotional states of self and other, (a) their judgment of the other person's emotions was shifted toward their own emotional state (EEB), and (b) the judgment of their own emotions was shifted toward the other's emotional state (EAB). The strength of these two biases was similar for behavioral pilot study 1 and the fMRI study, confirming our hypotheses that these biases are both relevant evidence for difficulties in self-other distinction. Additionally, both behavioral pilot study 2 and the fMRI study showed that the EEB was only observable during active emotional involvement in the game and not in the condition of passively observing the same game between four other players. In contrast to the two active conditions, passive observation led to more accurate, i.e., similar, judgments in both congruent and incongruent situations, possibly due to a lack of interference with one's own emotional state. In sum, our new version of Cyberball was able to produce feelings of social inclusion/exclusion and generated emotional biases to a similar extent compared with other paradigms who reported affective biases in other younger and adult neurotypical samples inducing social inclusion/exclusion (48) as well as for other emotions like envy or schadenfreude (50, 51), pleasant or unpleasant visuo-gustatory (22), visuo-tactile (23, 52–54), audio-visual (55) or face stimuli (56–58). Our results therefore extend previous findings on emotional alter- and egocentric biases to the domain of social emotions. Looking at behavioral group differences, we could not confirm our initial hypothesis of stronger emotional altercentric or egocentric biases in the autistic compared to the NT sample. Both biases were similarly high in the two groups, and significantly different from the passive condition, which is in line with the previous study indicating intact self-other distinction in autism (47).

On the neural level, the ROI analyses in rTPJ and rSMG revealed differences depending on the region, target, and group. In particular, we observed increased brain activity in rTPJ but not rSMG in neurotypical compared to autistic individuals when actively dealing with the EEB (compared to passively watching the ballgame). This indicates that individuals on the autism spectrum recruit rTPJ to a lesser extent than NT individuals when suppressing their own emotions to accurately judge the emotional state of another person, while this is not the case for rSMG. We further observed increased brain activity in rSMG but not rTPJ in neurotypical compared to autistic individuals when dealing with ego- compared to altercentric judgements. Here, a specific difference emerged when judging others compared to judging our own emotional states. Our results nicely complement and extend the findings reported in Hoffmann et al. (47), who observed reduced connectivity of regions of the Theory of Mind network (rTPJ) but an intact rSMG network connectivity during rest in autistic individuals. In line with the findings in that study, we show differential involvement of rTPJ and rSMG when dealing with conflicting emotional states in the same participant sample. This crucially underlines the necessity for a distinction between rSMG and rTPJ, and between ego- vs. altercentric biases, when investigating self-other distinction abilities in autism. They also crucially highlight that the type of control condition can lead to differential results. Future studies should focus on carefully teasing apart the separate roles of these two regions, for example, by employing more causal methods such as repetitive transcranial magnetic stimulation as in Silani et al. (23) and further investigate the best way to control for emotional ego- and altercentricity.

Generally, the brain results also showed that our intended manipulation of congruency regarding self- and other-related emotional states was successful. When actively playing the game and either judging their own or the other's incongruent emotional state, participants activated regions previously related to self-other distinction (59, 60), self/other attribution (61), conflict monitoring (62), mental imagery to represent the perspective of another person (63) and theory of mind (especially false-belief reasoning) as well as visual perspective taking [see (64–66) for meta-analyses]. Mentalizing abilities seem to be required more strongly in situations where two emotional states are incongruent compared to congruent (23). This was not the case in the passive viewing condition, where participants merely observed other individuals playing the Cyberball game. The active conditions thus recruited more cognitive self-other distinction processes than the passive condition, where participants had no own emotional state to keep track of.

In contrast to previous research, we did not specifically find increased brain activity in rSMG during incongruent compared to congruent judgements in our whole brain analyses. However, it should be noted that the crucial role of the rSMG in overcoming the EEB was found in a visuo-tactile paradigm (23) and a task using monetary rewards and punishment (50). Social emotions like in- or exclusion, on the other hand, belong to a different domain that could be processed in a different, possibly more cognitive way. Especially our task could have required more mentalizing abilities, as participants had to infer the emotional state of the other person who was displayed as an avatar, which might have recruited rTPJ more than rSMG.

In general, the observed neurophysiological group differences, despite similar behavior, demand further explanation. First of all, autism spectrum disorder is a very heterogeneous condition which may lead to a wide and diverse spectrum of social cognition abilities, ranging from neurotypical to different processing. As our sample was rather small, this diversity might have contributed to our findings. This crucially highlights the need for more implicit brain measures complementing subjective behavior, especially when investigating complex psychological phenomena such as social cognition (67, 68). In our case, different neural and computational processes in the brain might have led to similar behavior in both groups. Furthermore, as we mainly tested young and middle-aged adults, learning and adaptation processes could have contributed to intact self-other distinction abilities (69, 70). In this regard, it might be interesting to repeat our task in a younger population who had fewer opportunities to learn and adapt, to test directly whether this was the case. Lastly, the brain data represents activity during the relatively long blocks of playing Cyberball, while the behavioral ratings were collected at the end of each block. In other words, while the brain data might have been able to capture subtle differences in neural processing, the ratings might reflect an already corrected emotional perception. Future studies could clarify this issue by combining our here used task with behavioral measures of higher temporal resolution, such as a continuous rating of the emotional state over the whole playing time, or established physiological indicators of affect, such as pupil dilation or skin conductance response.

We would also like to briefly discuss and address the major strengths and limitations of the present study. Firstly, it has to be acknowledged that the two groups were closely matched regarding handedness, gender, age, general intelligence, and differed significantly in their autistic traits, but also differed in their level of reported depressive traits. This stands next to the fact that six of 21 autistic but no neurotypical participants indicated taking medication to treat e.g., depression and anxiety. As we did not explicitly assess comorbidities in our participants, we cannot fully disentangle possible interactions with medication intake or comorbidities. Future studies should therefore carefully assess the participants' medication history as well as other existing clinical diagnoses. Importantly, the higher levels of depression in the autistic group were very close to the cutoff for no to minimal depressive symptoms (35). Furthermore, the autistic group reported increased alexithymic traits compared to the NT group. This is not surprising, as higher alexithymic symptoms in individuals on the autism spectrum have been reported in multiple studies and alexithymia often co-occurs with autism (10, 71–73). However, including alexithymia as a covariate in our analyses did not change any of our here reported results. Furthermore, we found no associations in exploratory post-hoc analyses between the two emotional biases on the behavioral and brain level with trait depression or alexithymia, where we had observed group differences in the questionnaires (all *p*'s > 0.164).

Nevertheless, as Bird et al. (71) reported that the strength of left anterior insula activation in response to others' suffering was predictive of the degree of alexithymia in both autistic and NT groups but did not in fact vary as a function of group, future studies should better tease apart the proneness to emotional ego- and altercentric biases by specifically recruiting both NT and autistic individuals with high and low levels of alexithymia.

Secondly, the study used an adapted version of Cyberball with videos depicting real-life figures instead of cartoon animations. This was done to increase ecological validity and develop a task suitable for use in the autistic population that did not contain distractions such as background information, as the videos were edited to show white silhouettes on black background. The game also avoided solving the task by using emotional facial expressions, as all avatars kept a neutral expression in the videos. Furthermore, four confederates were introduced to the participant at the beginning of the session to create a realistic social situation and immerse the participants in the live gameplay. However, due to the length of the game and somewhat predictable nature of the conditions (i.e., the varying valence and congruence to measure EAB and EEB), it could well be that participants emotionally detached or disengaged from the game at some point and weren't able to empathize as strongly. However, the results of an increased behavioral EAB and EEB in the active but not the passive conditions in all our three studies, combined with the recruitment of mentalizing brain regions during the task in our fMRI study, showed that participants were still influenced by their own and the other person's emotions when they were actively engaged in the game. And as described by Zadro et al. (40), even ostracism by a computer compared to ostracism by real individuals is able to produce similar levels of social exclusion. Nevertheless, future studies using these types of social interaction setups should consider even stronger “interactive” playing modes that introduce greater variability and constantly remind the participant of the other players, e.g., *via* live videos.

Lastly, we did not include a passive condition for the altercentric bias, which could have made results regarding the EAB more informative. Future studies should therefore pay close attention to how the biases are being measured and include appropriate controls for all of them.

In conclusion, the present findings replicate previous behavioral and neurophysiological results on the ego- and altercentric biases in the emotional domain, but expand them to the field of social emotions. Our results suggest no behavioral differences in the processing of simultaneous emotional states and thus intact self-other distinction in individuals on the autism spectrum, but specific neurophysiological differences between autistic and non-autistic individuals rooted in rSMG and rTPJ when dealing with ego- and altercentric biases. This study has crucial implications for further research of social cognition abilities in autism. Investigating socio-emotional competences on a more basic level using valid paradigms and controls will ultimately pave the way to better understand individuals on the autism spectrum and could set a foundation for interventions promoting successful and long-lasting social interactions and relationships.

## Data Availability Statement

The original contributions presented in the study are included in the article/Supplementary Material, further inquiries can be directed to the corresponding author/s.

## Ethics Statement

The studies involving human participants were reviewed and approved by the Ethics Committee of the Medical University of Vienna (EK 1166/2015). The patients/participants provided their written informed consent to participate in this study.

## Author Contributions

HH: conceptualization, methodology, formal analysis, investigation, data curation, writing—original draft, writing—review and editing, visualization, project administration, and funding acquisition. LL: conceptualization, formal analysis, investigation, writing—original draft, and writing—review and editing. HHH: methodology, investigation, data curation, writing—review and editing, and funding acquisition. PS: conceptualization, methodology, investigation, data curation, and writing—review and editing. GS: conceptualization, methodology, software, investigation, resources, writing—original draft, writing—review and editing, supervision, project administration, and funding acquisition Brand et al. (74). All authors contributed to the article and approved the submitted version.

## Funding

This study was partially supported by a WWTF grant (CS15-003) to GS, and two promotional scholarships of the University of Vienna awarded to HH and HHH (https://studienpraeses.univie.ac.at/stipendien/foerderungsstipendien-nach-dem-studfg/). None of the funders had any role in study design, data collection and analysis, interpretation, writing or decision to publish.

## Conflict of Interest

HH works as a researcher and psychologist for MyMind GmbH, a company developing a neurofeedback training game for children with autism spectrum disorder and attention deficit hyperactivity disorder, but this work is in no way related to the present research. The remaining authors declare that the research was conducted in the absence of any commercial or financial relationships that could be construed as a potential conflict of interest.

## Publisher's Note

All claims expressed in this article are solely those of the authors and do not necessarily represent those of their affiliated organizations, or those of the publisher, the editors and the reviewers. Any product that may be evaluated in this article, or claim that may be made by its manufacturer, is not guaranteed or endorsed by the publisher.

## Footnotes

^1^The videos displayed male participants trained to show similar body posture during throwing and catching the ball, while the rhythm of the ball throws was clocked using a metronome. One person stood behind the camera catching and throwing the ball in front of the camera lens to create an egocentric perspective and viewed the other players standing to his/her left and right, and in front of the participant.

^2^To achieve these conditions, participants were ostensibly divided into two groups and informed that while Group A would only be able to throw to the left and right player, Group B would be able to throw to the left, right and front players. Participants were purposely always assigned to Group A, together with the opposite player. The deception was used to avoid participants throwing the ball to the opposite player after seeing him excluded by the other two.

^3^Note that in each behavioral pilot study only one control condition (either self active in behavioral pilot study 1 or other passive in behavioral pilot study 2) was implemented. In the fMRI study, both control conditions were employed.

## References

[1] Frith CD Frith U. Implicit and explicit processes in social cognition. Neuron. (2008) 60:503–10. 10.1016/j.neuron.2008.10.032 18995826

[2] Baron-Cohen S Tager-Flusberg H Lombardo MV. Understanding Other Minds: Perspectives From Developmental Social Neuroscience. (2013). Available online at: https://journals.google.com/journals?hl=deandlr=andid=3nloAgAAQBAJandpgis=1

[3] Singer T. The neuronal basis and ontogeny of empathy and mind reading: review of literature and implications for future research. Neurosci Biobehav Rev. (2006) 30:855–63. 10.1016/j.neubiorev.2006.06.011 16904182

[4] Happé F Cook JL Bird G. The structure of social cognition: in(ter)dependence of sociocognitive processes. Annual Rev Psychol. (2017) 68:243–67. 10.1146/annurev-psych-010416-044046 27687121

[5] de Vignemont F Singer T. The empathic brain: how, when and why? Trends Cogn Sci. (2006) 10:435–41. 10.1016/j.tics.2006.08.008 16949331

[6] Preckel K Kanske P Singer T. On the interaction of social affect and cognition: empathy, compassion and theory of mind. Curr Opin Behav Sci. (2018) 19:1–6. 10.1016/j.cobeha.2017.07.01033421860

[7] Reniers RLEP Völlm B Elliott R Corcoran R. Empathy, ToM, and self-other differentiation: an fMRI study of internal states. Social Neurosci. (2014) 9:50–62. 10.1080/17470919.2013.861360 24294841

[8] Thirioux B Mercier MR Blanke O Berthoz A. The cognitive and neural time course of empathy and sympathy: an electrical neuroimaging study on self-other interaction. Neuroscience. (2014) 267:286–306. 10.1016/j.neuroscience.2014.02.024 24583040

[9] American Psychiatric Association. (2013). Diagnostic and Statistical Manual of Mental Disorders (DSM-5) (5th ed.). New York, NY: American Psychiatric Publishing.

[10] Bird G Viding E. The self to other model of empathy: providing a new framework for understanding empathy impairments in psychopathy, autism, and alexithymia. Neurosci Biobehav Rev. (2014) 47:520–32. 10.1016/j.neubiorev.2014.09.021 25454356

[11] de Guzman M Bird G Banissy MJ Catmur C. Self-other control processes in social cognition: from imitation to empathy. Philosophic Transact Roy Soc B: Biol Sci. (2015) 371:20150079. 10.1098/rstb.2015.0079 26644597 PMC4685524

[12] Fan YT Chen C Chen SC Decety J Cheng Y. Empathic arousal and social understanding in individuals with autism: evidence from fMRI and ERP measurements. Soc Cogn Affect Neurosci. (2014) 9:8. 10.1093/scan/nst101 23929944 PMC4127026

[13] Gu X Eilam-Stock T Zhou T Anagnostou E Kolevzon A Soorya L. Autonomic and brain responses associated with empathy deficits in autism spectrum disorder. Hum Brain Mapp. (2015) 36:3323–38. 10.1002/hbm.22840 25995134 PMC4545680

[14] Deschrijver E Palmer C. Reframing social cognition: relational vs. representational mentalizing. Psychol Bull. (2020) 20:302. 10.1037/bul0000302 32852961

[15] Begeer S Bernstein DM Van Wijhe J Scheeren AM Koot HM. A continuous false belief task reveals egocentric biases in children and adolescents with Autism Spectrum Disorders. Autism. (2012) 16:357–66. 10.1177/1362361311434545 22399450

[16] Frith U de Vignemont F. Egocentrism, allocentrism, and Asperger syndrome. Conscious Cogn. (2005) 14:719–38. 10.1016/j.concog.2005.04.006 15996486

[17] Nijhof AD Shapiro KL Catmur C Bird G. No evidence for a common self-bias across cognitive domains. Cognition. (2020) 197:104186. 10.1016/j.cognition.2020.104186 31954993

[18] Tei S Fujino J Itahashi T Aoki Y Ohta H Kubota M. Egocentric biases and atypical generosity in autistic individuals. Autism Res. (2019) 19:1–11. 10.1002/aur.2130 31102339

[19] Dziobek I Rogers K Fleck S Bahnemann M Heekeren HR Wolf OT. Dissociation of cognitive and emotional empathy in adults with Asperger syndrome using the Multifaceted Empathy Test (MET). J Autism Dev Disord. (2008) 38:464–73. 10.1007/s10803-007-0486-x 17990089

[20] Mazza M Pino MC Mariano M Tempesta D Ferrara M de Berardis D. Affective and cognitive empathy in adolescents with autism spectrum disorder. Front Hum Neurosci. (2014) 8:791. 10.3389/fnhum.2014.00791 25339889 PMC4187579

[21] Hoffmann F Koehne S Steinbeis N Dziobek I Singer T. Preserved self-other distinction during empathy in autism is linked to network integrity of right supramarginal gyrus. J Autism Dev Disord. (2016) 46:637–48. 10.1007/s10803-015-2609-0 26476740

[22] Hoffmann F Singer T Steinbeis N. Children's increased emotional egocentricity compared to adults is mediated by age-related differences in conflict processing. Child Dev. (2015) 86:765–80. 10.1111/cdev.12338 25626453

[23] Silani G Lamm C Ruff CC Singer T. Right supramarginal gyrus is crucial to overcome emotional egocentricity bias in social judgments. J Neurosci. (2013) 33:15466–76. 10.1523/JNEUROSCI.1488-13.2013 24068815 PMC6618458

[24] Novembre G Zanon M Silani G. Empathy for social exclusion involves the sensory-discriminative component of pain: a within-subject fMRI study. Soc Cogn Affect Neurosci. (2015) 10:153–64. 10.1093/scan/nsu038 24563529 PMC4321615

[25] Williams KD Jarvis B. Cyberball: a program for use in research on interpersonal ostracism and acceptance. Behav Res Methods. (2006) 38:174–80. 10.3758/BF03192765 16817529

[26] Lord C Risi S Lambrecht L Cook EH Leventhal BL Dilavore PC. The Autism Diagnostic Observation Schedule—Generic: a standard measure of social and communication deficits associated with the spectrum of autism. J Autism Dev Disord. (2000) 30:205–23. 10.1023/A:1005592401947 11055457

[27] Faul F Erdfelder E Lang A-G Buchner A. G^*^Power 3: A flexible statistical power analysis program for the social, behavioral, and biomedical sciences. Behav Res Methods. (2007) 39:175–91. 10.3758/BF03193146 17695343

[28] Kratzmeier H Horn R. Standard Progressive Matrices (SPM). Test Manual. Beltz Test. (1988).

[29] Lehrl S. Mehrfachwahl-Wortschatz-Intelligenztest (MWT-B). PERIMED-spitta. (1995).

[30] Freitag CM Retz-Junginger P Retz W Seitz C Palmason H Meyer J. Evaluation der deutschen Version des Autismus-Spektrum-Quotienten (AQ)—die Kurzversion AQ-K. Zeitschrift Für Klinische Psychologie Und Psychotherapie. (2015) 36:280–9. 10.1026/1616-3443.36.4.28028686276

[31] Bach M Bach D de Zwaan M Serim M. Validierung der deutschen Version der 20-Item Toronto-Alexithymie-Skala bei Normalpersonen und psychiatrischen Patienten. PPmP: Psychotherapie. (1996) 96:208–214. 8850096

[32] Bagby RM Parker JDA Taylor GJ. The twenty-item Toronto Alexithymia scale-I. item selection and cross-validation of the factor structure. J Psychosomatic Res. (1994) 38:23–32. 10.1016/0022-3999(94)90005-1 8126686

[33] Davis MH. A multidimensional approach to individual differences in empathy. JSAS Catalog of Selected Documents in Psychology. (1980) 10:85.

[34] Paulus C. Ist die Bildung eines Empathiescores in der deutschen Fassung des IRI sinnvoll? (2012).

[35] Kühner PDC Bürger C Keller F Hautzinger M. Reliabilität und Validität des revidierten Beck-Depressionsinventars (BDI-II). Nervenarzt. (2007) 78:651–6. 10.1007/s00115-006-2098-7 16832698

[36] Lakens D. Calculating and reporting effect sizes to facilitate cumulative science: a practical primer for t-tests and ANOVAs. Front Psychol. (2013) 4:1–12. 10.3389/fpsyg.2013.00863 24324449 PMC3840331

[37] Abrams D Weick M Thomas D Colbe H Franklin KM. On-line ostracism affects children differently from adolescents and adults. Br J Dev Psychol. (2011) 29:110–23. 10.1348/026151010X494089 21288256

[38] Eisenberger NI Lieberman MD Williams KD. Does rejection hurt? An fMRI study of social exclusion. Science. (2003) 302:290–2. 10.1126/science.1089134 14551436

[39] Williams KD Cheung CK Choi W. Cyberostracism: effects of being ignored over the internet. J Pers Soc Psychol. (2000) 79:748–62. 10.1037/0022-3514.79.5.748 11079239

[40] Zadro L Williams KD Richardson R. How low can you go? Ostracism by a computer is sufficient to lower self-reported levels of belonging, control, self-esteem, and meaningful existence. J Exp Soc Psychol. (2004) 40:560–7. 10.1016/j.jesp.2003.11.006

[41] Mathworks. MATLAB (R2010*b*). (2010).

[42] Rife SC Nuijten MB Epskamp S. statcheck: extract statistics from articles recompute p-values [web application]. (2016). Available online at: Retrieved from http://statcheck.io

[43] R Core Team (2020). R: A Language and Environment for Statistical Computing. R Foundation for Statistical Computing. Available online at: https://www.r-project.org/

[44] Duff EP Cunnington R Egan GF. REX: response exploration for neuroimaging datasets. Neuroinformatic. (2007) 5:223–34. 10.1007/s12021-007-9001-y 17985253

[45] Eickhoff SB Stephan KE Mohlberg H Grefkes C Fink GR Amunts K. A new SPM toolbox for combining probabilistic cytoarchitectonic maps and functional imaging data. Neuroimage. (2005) 25:1325–35. 10.1016/j.neuroimage.2004.12.034 15850749

[46] Brett M Romain JL Valabregue A Jean-Baptiste P. “Region of interest analysis using an SPM toolbox [abstract],” In: Presented at the 8th International Conference on Functional Mapping of the Human Brain, June 2-6, Sendai, Japan. (2002). Available on CD-ROM in NeuroImage.

[47] Hoffmann F Banzhaf C Kanske P Gärtner M Bermpohl F Singer T. Empathy in depression: egocentric and altercentric biases and the role of alexithymia. J Affect Disord. (2016) 199:23–9. 10.1016/j.jad.2016.03.007 27060429

[48] Seidel E-M Silani G Metzler H Thaler H Lamm C Gur RC. The impact of social exclusion vs. inclusion on subjective and hormonal reactions in females and males. Psychoneuroendocrinology. (2013) 38:2925–32. 10.1016/j.psyneuen.2013.07.021 23972943 PMC3863951

[49] Wang H Braun C Enck P. How the brain reacts to social stress (exclusion)–a scoping review. Neurosci Biobehav Rev. (2017) 5:12. 10.1016/j.neubiorev.2017.05.012 28535967

[50] Steinbeis N Bernhardt BC Singer T. Age-related differences in function and structure of rSMG and reduced functional connectivity with DLPFC explains heightened emotional egocentricity bias in childhood. Soc Cogn Affect Neurosci. (2014) 10:302–10. 10.1093/scan/nsu057 24771281 PMC4321629

[51] Steinbeis N Singer T. Projecting my envy onto you: neurocognitive mechanisms of an offline emotional egocentricity bias. Neuroimage. (2014) 102:370–80. 10.1016/j.neuroimage.2014.08.007 25117603

[52] Riva F Triscoli C Lamm C Carnaghi A Silani G. Emotional egocentricity bias across the life-span. Front Aging Neurosci. (2016) 8:1–7. 10.3389/fnagi.2016.00074 27199731 PMC4844617

[53] Tomova L Von Dawans B Heinrichs M Silani G Lamm C. Is stress affecting our ability to tune into others? evidence for gender differences in the effects of stress on self-other distinction. Psychoneuroendocrinology. (2014) 43:95–104. 10.1016/j.psyneuen.2014.02.006 24703175

[54] Riva F Lenger M Kronbichler M Lamm C Silani G. Age-related changes in human emotional egocentricity: evidence from multi-level neuroimaging. BioRxiv. (2019) 12:784215. 10.1101/784215

[55] von Mohr M Finotti G Ambroziak KB Tsakiris M. Do you hear what I see? an audio-visual paradigm to assess emotional egocentricity bias. Cogn Emot. (2020) 34:756–70. 10.1080/02699931.2019.1683516 31672095

[56] Naor N Shamay-Tsoory SG Sheppes G Okon-Singer H. The impact of empathy and reappraisal on emotional intensity recognition. Cogn Emot. (2018) 32:972–87. 10.1080/02699931.2017.1372366 28891381

[57] Trilla I Eiserbeck F Dziobek I. Projecting the own affective states onto others: no influence of perceived similarity. Preprint. (2020) 20:4. 10.31234/osf.io/j2ct4

[58] Trilla I Weigand A Dziobek I. Affective states influence emotion perception: evidence for emotional egocentricity. Psychol Res. (2020) 0123456789:1. 10.1007/s00426-020-01314-3 32206856 PMC8049894

[59] Lamm C Bukowski H Silani G. From shared to distinct self-other representations in empathy: evidence from neurotypical function and socio-cognitive disorders. Philosophic Trans Roy Soc London Series B, Biol Sci. (2016) 371:20150083. 10.1098/rstb.2015.0083 26644601 PMC4685528

[60] Steinbeis N. The role of self–other distinction in understanding others' mental and emotional states: neurocognitive mechanisms in children and adults. Philosophic Trans Roy Soc London Series B, Biol Sci. (2016) 371:20150074. 10.1098/rstb.2015.0074 26644593 PMC4685520

[61] Kestemont J Ma N Baetens K Clément N Overwalle F Van. Neural correlates of attributing causes to the self, another person and the situation. Soc Cogn Affect Neurosci. (2015) 10:114–21. 10.1093/scan/nsu030 24633532 PMC4994850

[62] Jeannerod CM. A causal role for right temporo-parietal junction in signaling moral conflict. Elife. (2018) 12:1–42. 10.7554/eLife.40671 30561334 PMC6298767

[63] Cavanna AE Trimble MR. The precuneus: a review of its functional anatomy and behavioural correlates. Brain. (2006) 129:564–83. 10.1093/brain/awl004 16399806

[64] Molenberghs P Johnson H Henry JD Mattingley JB. Understanding the minds of others: a neuroimaging meta-analysis. Neurosci Biobehav Rev. (2016) 65:276–91. 10.1016/j.neubiorev.2016.03.020 27073047

[65] Schurz M Aichhorn M Martin A Perner J. Common brain areas engaged in false belief reasoning and visual perspective taking: a meta-analysis of functional brain imaging studies. Front Hum Neurosci. (2013) 7:1–14. 10.3389/fnhum.2013.00712 24198773 PMC3814428

[66] Schurz M Radua J Tholen MG Maliske L Margulies DS Rogier B. Toward a hierarchical model of social cognition: a neuroimaging meta-analysis and integrative review of empathy and theory of mind. Psychol Bull Adv. (2020) 12:303. 10.1037/bul0000303 33151703

[67] Chiel HJ Beer RD. The brain has a body: adaptive behavior emerges from interactions of nervous system, body and environment. Trends Neurosci. (1997) 20:553–7. 10.1016/S0166-2236(97)01149-1 9416664

[68] Kriegeskorte N Douglas PK. Cognitive computational neuroscience. Nat Neurosci. (2018) 21:1148–60. 10.1038/s41593-018-0210-5 30127428 PMC6706072

[69] Frith CD Frith U. Mechanisms of social cognition. Annual Review of Psychol. (2011) 63:287–313. 10.1146/annurev-psych-120710-100449 21838544

[70] Kramer DA. A life-span view of social cognition. Educational Gerontol. (2006) 12:277–89. 10.1080/0380127860120403

[71] Bird G Silani G Brindley R White S Frith U Singer T. Empathic brain responses in insula are modulated by levels of alexithymia but not autism. Brain. (2010) 133:1515–25. 10.1093/brain/awq060 20371509 PMC2859151

[72] Bird G Cook R. Mixed emotions: the contribution of alexithymia to the emotional symptoms of autism. Transl Psychiatry. (2013) 3:7. 10.1038/tp.2013.61 23880881 PMC3731793

[73] Chester DS Pond RS Nathan DeWall C. Alexithymia is associated with blunted anterior cingulate response to social rejection: implications for daily rejection. Social Cognitive and Affective Neurosci. (2013) 10:517–22. 10.1093/scan/nsu082 24894765 PMC4381235

[74] Brand AE Allen L Altman M Hlava M. Beyond authorship: attribution, contribution, collaboration, and credit. Learned Publishing. (2015) 28:151–5. 10.1087/2015021120150211

